# Temperature Assisted in-Situ Small Angle X-ray Scattering Analysis of Ph-POSS/PC Polymer Nanocomposite

**DOI:** 10.1038/srep29917

**Published:** 2016-07-20

**Authors:** Ramdayal Yadav, Minoo Naebe, Xungai Wang, Balasubramanian Kandasubramanian

**Affiliations:** 1 Deakin University, Institute for Frontier Materials (IFM), Geelong, Australia; 2 Department of Materials Engineering, Defence Institute of Advanced Technology, Ministry of Defence, Girinagar, 411025 Pune, India

## Abstract

Inorganic/organic nanofillers have been extensively exploited to impart thermal stability to polymer nanocomposite via various strategies that can endure structural changes when exposed a wide range of thermal environment during their application. In this abstraction, we have utilized temperature assisted *in-situ* small angle X-ray scattering (SAXS) to examine the structural orientation distribution of inorganic/organic nanofiller octa phenyl substituted polyhedral oligomeric silsesquioxane (Ph-POSS) in Polycarbonate (PC) matrix from ambient temperature to 180 °C. A constant interval of 30 °C with the heating rate of 3 °C/min was utilized to guise the temperature below and above the glass transition temperature of PC followed by thermal gravimetric, HRTEM, FESEM and hydrophobic analysis at ambient temperature. The HRTEM images of Ph-POSS nano unit demonstrated hyperrectangular structure, while FESEM image of the developed nano composite rendered separated phase containing flocculated and overlapped stacking of POSS units in the PC matrix. The phase separation in polymer nanocomposite was further substantiated by thermodynamic interaction parameter (χ) and mixing energy (E_mix_) gleaned via Accelrys Materials studio. The SAXS spectra has demonstrated duplex peak at higher scattering vector region, postulated as a primary and secondary segregated POSS domain and followed by abundance of secondary peak with temperature augmentation.

The exceptional mechanical, physical, thermal and magnetic properties of inorganic/organic polymer hybrid nanocomposites have attracted great attention in recent years^1^,^2^,^3^,^4^,^5^. Numerous organo-fuctionalized compound based on silica, silicates, transition metal oxides, carbon, or other inorganic cores have been developed^6^. Among various organic/inorganic nano building block, polyhedral oligomeric silsesquioxane (POSS) has been recognized for its ability to impart thermal stability in polymer composite^7^. POSS can be regarded as the smallest silica particle (1–3 nm) with hybrid organic-inorganic nature, whose inorganic core imparts molecular reinforcement while its functionalized organic moieties provide schemes for reaction and compatibilization with the host polymer^8^,^9^. The flexible chemistry of POSS renders wide variety of substituents to the silica core and enhanced compatibility is expected when POSS substituents and polymer matrix contain similar moieties^10^. POSS has been widely incorporated with polyurethanes, polyethylene, polystyrene, poly (methyl methacrylate), polycarbonate and thermosets via various technique like copolymerization, grafting, blending and solvent casting^11^,^12^,^13^,^14^,^15^,^16^,^17^,^18^,^19^,^20^.

In order to develop thermally stable and flame retardant materials, polycarbonate (PC) has allured great interest due to its high tendency of charring during combustion^21^. However, further improvement in thermal stability has been achieved by incorporating functionalized POSS using various techniques like copolymerization, melt blending and solvent casting^10^,^22^. Though, copolymerization has been effectively employed to yield elevation in flammability, thermal or mechanical property, blending is recognized as inexpensive and simple approach for industrial scale application^23^,^24^. For instance, Song *et al*. have demonstrated the enhancement in thermal degradation and combustion behavior of PC/trisilanolphenyl POSS nanocomposite via melt blending while Hao *et al*. have elucidated the incorporation of phenethyl POSS in polycarbonate matrix by solvent casting technique^10^,^25^. The discrepancy in the dispersion level and POSS compatibility has been attributed to the preferential interaction between polymer matrix and POSS moieties during melt blending. Such interaction has been found limited in solvent casting due to the phase separation.

Although, the incorporation of POSS in polycarbonate has been investigated with polycarbonate for applications ranging from heat sink to the flame retardant materials, there is little literature reporting on *in-situ* quantification of structural orientation distribution of POSS nanoparticles in polymeric composite systems. Molecular interaction and interplay can be examined by temperature assisted *in-situ* SAXS analysis. In this study, we have attempted to quantitatively evaluate the molecular interaction and interplay between the intercalated POSS layers in the polycarbonate matrices via temperature assisted small angle x-ray scattering, thermal gravimetric analysis, FESEM and hydrophobic analysis at ambient temperature. We have also proposed the possible mechanism involved in structural changes under the various temperatures. In order to contemplate the phase separation due to high loading of POSS nano unit in PC matrix, the dynamic molecular simulation was accomplished for computing the dynamic thermal interaction and mixing energy at various temperatures. Further, we have speculated that the formulation of free volume in PC matrix enacted pronounced effect on molecular distribution and interplay over the hyperrectangle POSS at a wide range of temperature in PC matrix.

## Materials and Experimental Details

**Figure 1:**
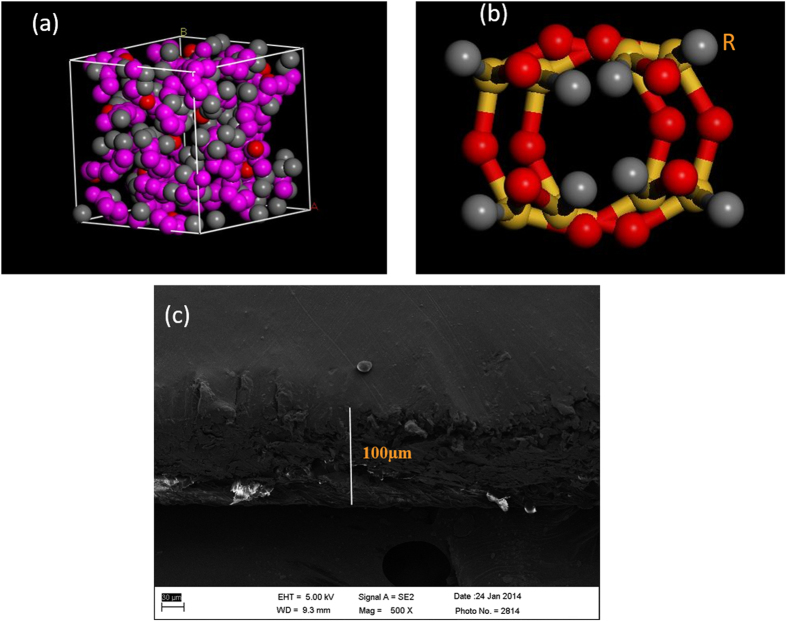
(**a**) Force field Optimized Amorphous Unit Cell of PC constructed by Materials Studio. **(b)** Atomistic representation of single POSS molecule by Materials Studio where R = Phenyl group. (**c**) Illustration of developed nanocomposite polymer thickness by Cross-Section FESEM of the polymer nanocomposite.

Polycarbonate (MFI = 10.5 g/10 min, viscosity 22 cp, LEXAN grade 143R, SABIC Innovative Plastics India Pvt. Ltd, India), Octaphenyl (OP) substituted POSS and Chemicals viz. Chloroform (Sigma Aldrich Corp. USA.) were used for the preparation of samples. All reagents and solvents involved were of analytical grade and were used without any further purification. The optimized amorphous unit cell of PC has been constructed by utilizing forcefield module of Materials Studio as shown in Fig. 1(a) while 3-D atomistic model of POSS has been delineated in Fig. 1(b). The grade of the polycarbonate utilized in this study is the amorphous grade with the melt temperature of 293 °C.

Solvent casted PC/Ph-POSS nanocomposite films were fabricated by dissolving 20 wt % of polycarbonate in chloroform followed by incorporating 10 to 30 wt % of Ph-POSS in the prepared polymer solution. The solution was then treated by ultrasonication for 5 min and subsequently casted on a glass slide in closed chamber to avoid abrupt evaporation of solvent. The detached polymer nanocomposite was dried under the vacuum at the boiling temperature of chloroform (61 °C) for 24 hr to remove retained traces of solvent. The maximum loading of Ph-POSS in PC matrix was ascertained to be 30 wt %, beyond this concentration the formation “pin hole” was observed under the scanning electron microscopy for the film thickness of approximately 100 μm as elucidated in Fig. 1(c).

## Results and Discussions

**Figure 2:**
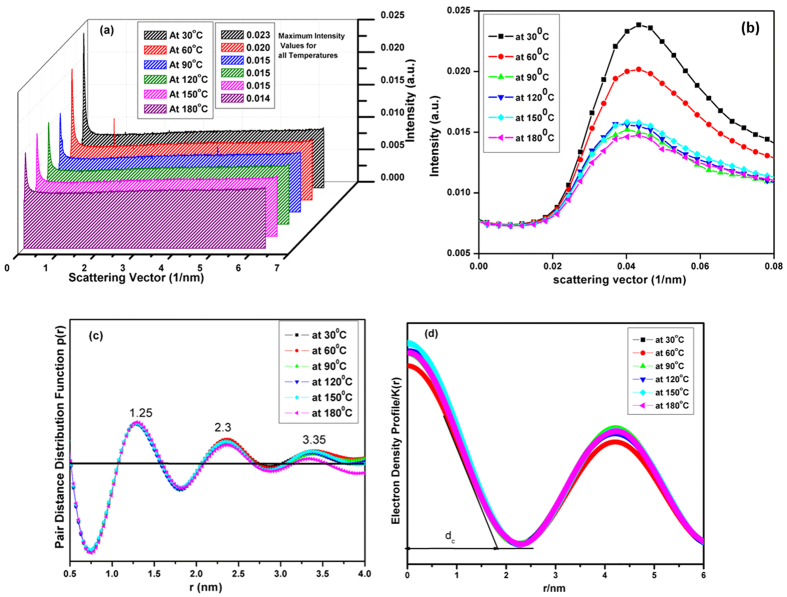
SAXS spectra of pristine PC at various temperature. (**a**) Full scattering curve (**b**) Scattering curve for lower q region (**c**) Pair distance correlation function for pristine PC at various (**d**) Electron Density correlation function for Pristine PC.

It has been ascertained that, the polymeric chain in a solvent cast film can preferentially align in a plane of casting substrate due to the freezing of molecular conformation into the film during the drying process^26^,^27^. Prest *et al*. demonstrated that the evaporation of volatile solvent caused development of entangled polymer chain by the viscosity upturn of the polymer solution followed by film disruption which rendered, unorganized and aggregated structural domain^28^. These localized aggregation of structural domains presumably lead to the development of partial orderliness as shown in SAXS spectra of PC at ambient temperature in Fig. 2(a). The temperature assisted *in-situ* small angle X-ray pattern of pristine PC at various temperatures has been exemplified in Fig. 2(a,b). In instance, for all the temperature, the first peak can be associated with the lamellar periodicity produced by those entangled domains and imperfect crystals of polymeric matrix^29^. As shown in Fig. 2(b), the intensity of the SAXS spectra was contemplated to be exceptionally reduced till 90 °C which can be successfully explained by the hypothesis of the Siegmann *et al*. Siegmann *et al*. have annotated that all solution casted PC thin film retains a small amount of solvent and this residual solvent (less than 1%) is sufficient to affect molecular structure of the film^30^. Therefore, the increase in the temperature results in the disruption of redundant excess free volume formed during the solvent evaporation due to the high degree of molecular mobility when the film was unveiled to the critical boiling temperature of chloroform (61 °C). The molecular mobility of film continues to the 90 °C till all the retained solvent was not completely exhausted from the film. Beyond this temperature we have observed partial change in peak intensity with the constant scattering vector at 0.041 nm. Further, pair distance distribution function was obtained from generalized indirect Fourier transformation technique. The interatomic distances present in the sample and each pair of the molecules accord rise to a peak as shown in Fig. 2(c) 31,32. Figure 2(c) shows three correlation peaks corresponding to the existence of three neighboring molecules at a distance of 1.25 nm, 2.3 nm and 3.35 nm within the maximum distance of 3.75 nm. Bandhopadhyay *et al*. demonstrated that, the presence of maxima and minima in pair correlation function was likely due to overlapping of the neighboring domains^33^. The unaltered correlation maxima for all the temperatures may be the unchanged average intermolecular distance, independent of the disruption of the aggregated domain in the presence of retained solvent.

Polycarbonate has been regarded as crystallizable amorphous polymer with granular structure that can be affected by thermal and mechanical treatment below glass transition temperature^34^,^35^,^36^,^37^. However, in contrast to the crystallizable polymers, the process of crystallization in polycarbonate is very slow. In our study, we are expecting no change in the amorphous character of PC during its exposure to the temperature assisted SAXS spectra. In order to enumerate the temperature dependence crystal/amorphous ratio during SAXS spectra, electron density correlation function has been employed in conjunction with Bragg’s law as illustrated in eqs 1 and 2 respectively^38^. This methodology was utilized by Men *et al*. to calculate linear crystallinity of cold-drawn polyethylene^38^,^39^. It must be noted that it is impossible to decide whether the deduced thickness is amorphous or crystalline obtained from correlation function without prior knowledge of the crystallinity.


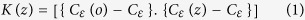



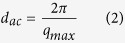


where K(z) = one dimensional correlation function, Ce(z) is electron density distribution along the stack normal, d_ac_ is long spacing obtained from Brag’s law (d_ac_ = d_a _+ d_c_, where d_a_ and d_c_ are the average thickness of amorphous and crystalline region as shown in Fig. 2d).

The inter-lamellar distance or long spacing values for all the temperature (from 30 °C to 150 °C) obtained from Brag’s law and electron density correlation function are indexed in Table 1. As evidenced form Table 1, the amorphous regions were not found to be altered significantly and it has been postulated that the crystalline region marked in electron density correlation function is very small to demonstrate the effect of crystallite in surrounding amorphous region^38^. Therefore, we have postulated that there is no significant change in the amorphousity of PC during the *in-situ* temperature assisted SAXS spectra.

**Figure 3:**
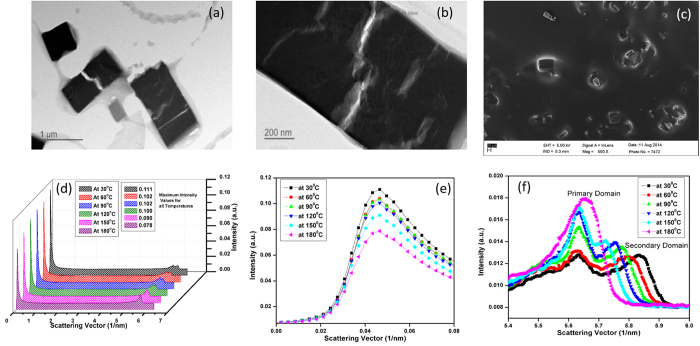
(**a**) TEM image of the single POSS unit demonstrating hyper-rectangle structure (**b**) HRTEM image of the single POSS moiety. (**c**) FESEM images of polymer Ph-POSS/PC nanocomposite (**d**) Full scattering pattern for polymer nanocomposite (PC/POSS) for various temperature (**e**) Scattering pattern for lower q region, exemplifying lamellar periodicity of PC at lower q region (**f**) SAXS pattern delineating primary and secondary aggregates at various temperature (higher q region).

The work of Zheng *et al*. have demonstrated that octa substituted POSS nanoparticles with various end groups (hydrogen, ethyl, methyl, isopropyl, cyclohexyl or phenyl) form rhombohedral (equivalent hexagonal crystal structure with similar X-ray fingerprints^40^,^41^. These cubical silsesquioxane can be regarded as nearly spherical unit which are arranged in a plane on a hexagonal array. The HRTEM image of the stacked POSS units has been shown in Fig. 3(a,b). POSS containing polymer nanocomposite acquire high thermal and mechanical stability either from aggregates of POSS nano units into the larger clusters or the degree of compatibility with the host polymer components^41^. In agreement, mechanically reinforced Ph-POSS nano units in PC polymer matrix has been elucidated in Fig. 3(c) which demonstrates flocculated, overlapping POSS nano units in various domains. We have also perceived analogous results in SAXS pattern of the developed polymer nanocomposite. In contrast to the SAXS spectra of PC, the stacking of POSS units form crystalline aggregates in the polymer matrix, can be clearly observed in the SAXS spectra as shown in Fig. 3(d,e). Figure 3(d) exhibits the enhancement in the SAXS intensity at lower q region resulting from crystalize POSS aggregates which has been also illustrated in Table 2.

According to the Gibbs phase rule, there are only two possible phases at constant temperature and pressure. Therefore, we have annotated that the incorporation of utmost possible POSS loading in 100 μm thin film accorded non uniform distribution of POSS units and formed flocculated clusters with two different phases (POSS rich domain and PC rich phase). In addition, the duplex peak in SAXS spectra of nanocomposite at higher q region (Fig. 3f) has been interpreted as peak obtained from primary domain and secondary domain. As stated earlier, the evaporation of the volatile solvent can cause the localized aggregation of the structural domain which presumably lead to the development of primary and secondary domain observed in PC/POSS composite as shown in Fig. 3(d).

As annotated by Prest *et al*. as solvent evaporates, the molecules of the film reduce their ability to respond the increased relaxation time^26^. Further evaporation of the solvent locks out the molecules at their respective positons and they are no longer able to relax to their new equilibrium state. Based on this postulation, we have concluded that primary domains are associated with those aggregates which are locked in the matrix with the higher concentration. While secondary domains correspond to the lower concentration of POSS unit with the reduced inter lamellar stacking as delineated in Table 2. SAXS spectra of the primary domains renders intensified and stable peak with constant inter lamellar distance of 11.14 Å till 150 °C while the alteration of secondary peak followed by its abundance with temperature augmentation was noticed for secondary domain. As stated earlier, polymer nanocomposite contains various domains, coined as primary and secondary domain whose temperature assisted conducts has been substantially controlled by available free volume in both the domains for given volume at any instant. In this context, we have contemplated that primary domains are dominated by POSS rich phase and secondary POSS domains are dominated by PC rich phase. In the consequence of increasing temperature from ambient to 60 °C, we have postulated intercalation of PC moieties in stacked hyper-rectangle POSS units resulting in enhanced inter lamellar distance in contrast to consistent primary aggregates. Further increased temperature i.e. 90 °C can be assigned as a critical temperature for developed polymer nanocomposite because at this temperature we have discerned intensified SAXS spectra for primary aggregates, speculated to the partial reduction in interfacial layer that continues to120 °C and endowed to be constant till 150 °C. While disruption of secondary domain extended till 150 °C by the virtue of continuous intercalation of PC unit in stacked POSS moieties. It is important to note that, diminished SAXS spectra at lower scattering vector region was ascertained beyond 120 °C as elucidate in Fig. 3(f). This phenomenon can be associated to the thermally assisted randomization of PC rich domains due to the co-operative movement of the phenyl group with the molecular motion of the carbonyl group^42^. Therefore, secondary domain undergoes complete disruption and fragmentation with the reduced extractability under the SAXS spectra at 180 °C due to the segmental movement of the polymer matrix without altering primary domain. At the 180 °C temperature we have noticed single spectra at higher scattering vector region with reduced inter lamellar distance due to the complete disruption of secondary domains.

**Figure 4:**
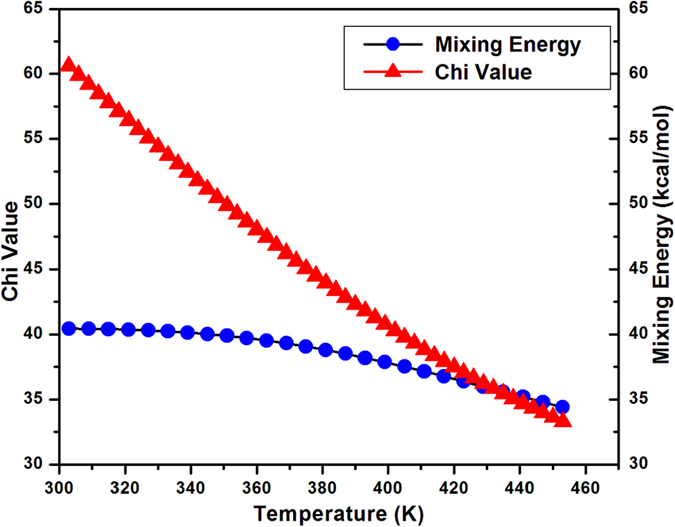
Computation of Thermodynamic Interaction Parameter (χ ) and Mixing Energy (E_mix_) via Materials Studio.

In order to substantiate the phase separation in polymer nanocomposite, thermodynamic interaction parameter or solubility parameter (χ) and mixing energy (E_mix_) has been delineated via Materials Studio that utilizes off lattice method which combined extended Flory Huggins model and molecular simulation technique. Utracki has evinced that the conversion of room temperature to high temperature impart the effect of decreasing solubility parameter^43^,^44^. We have obtained analogous result of solubility parameter with the increase in the temperature as exemplified in Fig. 4. It has been ascertained that the positive χ and E_mix_ value obtained at ambient temperature allied to the phase separation as conferred in SAXS spectra of polymer nanocomposite previously while reduction in the value with the amelioration in temperature relinquish some extent of intercalation or mixing^45^,^46^.

**Figure 5:**
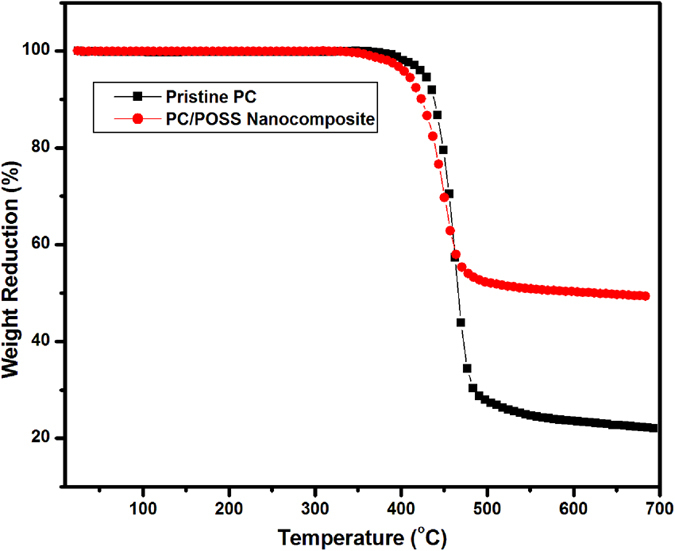
Thermal gravimetric analysis of pristine PC and Ph-POSS/PC nanocomposite.

Thermal stability and thermal oxidative degradation of the nacreous composite were delineated by thermal gravimetric analysis as shown in Fig. 5. The obtained result reveals that the initiation of thermal decomposition and temperature at maximum decomposition was eloquently improved in nano Ph-PC/POSS polymer nanocomposite by the formation of silicon containing heat shield layer a high temperature. In this framework, the suppressed mass loss was imputed by uncovering of the entrapped nano asperities of POSS by the complete mass loss of PC matrix followed by conversion of POSS in silicates after degradation^47^,^48^,^49^,^50^. The suppressed mass loss can also be ascertained by the ordered distribution of POSS in PC lamellae space which yielded shielding effect until the external temperature is high enough to the melting point of the polymer matrix which further concluded by the calculation of latent heat of fusion.

**Figure 6:**
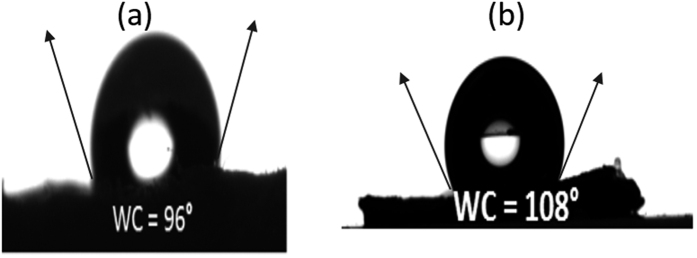
Contact angle measurement for (**a**) pristine PC and (**b**) Ph-POSS/PC composite.

The hydrophobic characteristics of nanocomposite were outlined by measuring the contact angle of the sessile drop based on the apprehension developed by Balasubramanian *et al*. They have extensively explored the contact angle of various surfaces which predominantly depends on the surface roughness, surface energy and surface tension based on the proposed model of Cassie and Baxter^51^,^52^,^53^. In this context, we have contemplated hydrophobic attribute of developed polymer nano composite based on alteration in surface energy by the amalgamation of POSS units in PC matrix. Mishra *et al*. in their abstraction manifested that increasing POSS content displayed the ability to reduce the surface energy of the polymer nanocomposite^54^. We have noticed analogous effect when Ph-POSS was incorporated in PC matrix, which yield reduction (surface energy of pristine PC- 32 ± 2 mN/m and for nanocomposite 23 ± 3 mN/m) in surface energy, contributing to the enhancement in contact angle by 11% of enhanced contact angle as demonstrated in Fig. 6(a,b). The peculiarity of hydrophobic surface is commonly interpreted via the hypothesis of Cassie-Baxter state and Wenzel state intended by Wenzels and Cassie–Baxter. Cassie-Baxter state unravel the state in which the liquid does not penetrate hallows of corrugated surface while Wenzel state exist when liquid is in contact with entire exposed surface of the solids^55^,^56^,^57^. In disparity to aforementioned hypothesis, we assumed that polymer nanocomposite in our abstraction pursue the perception of Gao *et al*., which report that the contact angle of sessile drop on solid surface depends on the three phase contact line formed due to the perpetuation of the drop on surface^58^,^59^,^60^,^61^. As previously stated, polymer nanocomposite encompasses primary and secondary aggregated domain in PC matrix, therefore, we envision that instead of conceding the formation of Cassie-Baxter and Wenzel state, the sessile drop will form the measurable contact by the virtue of contact line pinning in the presence of stacked POSS units.

## Conclusions

In this contemplation, we have delineated the temperature assisted *in-situ* SAXS spectra of solvent casted phenyl substituted POSS/Polycarbonate nanocomposite for first time to deduce the possible structural changes and interplay of POSS moieties in PC matrix. We have observed the formation of duplex peak at higher scattering vector region which was associated to the strongly bonded primary domain and loosely bonded secondary domain containing various amount of free volume via stacking of hyper-rectangle POSS unit. The increase in the local free volume in polymer nanocomposite portrayed indispensable conduct under the advancement of temperature. The secondary peak was found to be abundant and un-extractable with temperature augmentation during temperature assisted *in-situ* SAXS analysis that is presumably assumed for those clusters which contains sizable free volume by stacking of large number of hyper-rectangular POSS units. As expected, we have contemplated improved thermal stability of the polycarbonate film by incorporating POSS nano units in the matrix. We envision that the hydrophobic attribute of the polymer nanocomposite presumably follows the Gao’s hypothesis instead of conceding the formation of Cassie-Baxter and Wenzel state.

## Additional Information

**How to cite this article**: Yadav, R. *et al*. Temperature Assisted in-Situ Small Angle X-ray Scattering Analysis of Ph-POSS/PC Polymer Nanocomposite. *Sci. Rep*. **6**, 29917; doi: 10.1038/srep29917 (2016).

## Figures and Tables

**Table 1:** Illustration of long spacing value obtained from Bragg’s law and one dimensional correlation function.

Temperature	Long Spacing (d_ac_) calculated from Bragg’s Law (nm)	The average thickness of Amorphous Region (d_a_)	Amorphous Region (d_a_/d_ac_ in %)
30 °C	139.6	137.6	98.56
60 °C	146.1	144.1	98.63
90 °C	153.2	151.2	98.69
120 °C	153.2	151.2	98.69
150 °C	153.2	151.2	98.69
180 °C	153.2	151.2	98.69

**Table 2:** The intensity comparison between pristine PC and PC/POSS polymer nanocomposite at lower q region.

Temperature (°C)	Intensity for Pristine PC	Intensity for Pristine PC/POSS nanocomposite
30	0.023	0.111
60	0.022	0.102
90	0.015	0.102
120	0.015	0.100
150	0.015	0.09
180	0.014	0.078

## References

[b1] Okada A. & Usuki A. The Chemistry of Polymer-clay Hybrids. Mater. Sci Eng. C 3(2), 109–15 (1995).

[b2] Gilman J. W. Flammability and Thermal Stability Studies of Polymer Layered-silicate (clay) Nanocomposites. Appl. Clay Sci. 15, 31–49 (1999).

[b3] Gilman J. W. . Flammability Properties of Polymer−Layered-Silicate Nanocomposites. Polypropylene and Polystyrene Nanocomposites. Chem. Mater. 12(7), 1866–1873 (2000).

[b4] Porter D., Metcalfe E. & Thomas M. J. K. Nanocomposite Fire Retardants - A Review. Fire Mater. 24, 45–52 (2000).

[b5] Zanetti M., Lomakin S. & Camino G. Polymer Layered Silicate Nanocomposites. Macromol. Mater Eng. 279(1), 1–9 (2000).

[b6] Monticelli O., Fina A., Ullah A. & Waghmare P. Preparation, Characterization, and Properties of Novel PSMA-POSS Systems by Reactive Blending. Macromolecules 42, 6614–6623 (2009).

[b7] Schwab J. J. & Lichtenhan J. D. Polyhedral Oligomeric Silsesquioxane (POSS)-Based Polymers. Appl. Organomet. Chem. 12, 707–713 (1998).

[b8] Thomas S. & Stephen R. Rubber Nanocomposite Preparation, Properties and Application Vol. 1, Ch. 1, 1–20 (John Wiley and Sons (Asia) Pvt Ltd., 2010).

[b9] Milliman H. W., Ishida H. & David A. S. Structure Property Relationships and the Role of Processing in the Reinforcement of Nylon 6-POSS Blends. Macromolecules 45, 4650–4657 (2012).

[b10] Hao N., Bolhning M., Goering H. & Scholnhals A. Nanocomposites of Polyhedral Oligomeric Phenethylsilsesquioxanes and Poly (bisphenol A carbonate) as Investigated by Dielectric Spectroscopy. Macromolecules 40(8), 2955–2964 (2007).

[b11] Fu B. X. . Nanoscale Reinforcement of Polyhedral Oligomeric Silsesquioxane (POSS) in Polyurethane Elastomer. J. Polym. Int. 49(5), 437 (2000).

[b12] Zheng L., Kasi R. M., Farris R. J. & Coughlin E. B. Copolymers of Polyethylene, Polypropylene, and Polystyrene Containing Polyhedral Oligomeric Silsesquioxane (POSS). ACS, Division of Polymeric Materials: Science and Engineering 84, 114–115 (PMSE Preprints 2001).

[b13] Zheng L., Kasi R. M., Farris R. J. & Coughlin E. B. Synthesis and Thermal Properties of Hybrid Copolymers of Syndiotactic Polystyrene and Polyhedral Oligomeric Silsesquioxane. J. Polym. Sci., Part A: Polym. Chem. 40(7), 885 (2002).

[b14] Patel R. R., Mohanraj R. & Pittman C. U. Jr. Properties of Polystyrene and Polymethyl methacrylate Copolymers of Polyhedral Oligomeric Silsesquioxanes: A Molecular Dynamics Study. J. Polym. Sci., Part B: Polym. Phys. 44, 234 (2006).

[b15] Ni Y. & Zheng S. Epoxy Resin Containing Octamaleimidophenyl Polyhedral Oligomeric Silsesquioxane. Macromol. Chem. Phys. 206(20), 2075 (2005).

[b16] Li S., Simon G. P. & Matisons J. G. The Effect of Incorporation of POSS Units on Polymer Blend Compatibility. J. Appl. Polym. Sci. 115(2), 1153 (2010).

[b17] Wu J., Haddad T. S., Kim G. & Mather P. T. Rheological Behavior of Entangled Polystyrene−Polyhedral Oligosilsesquioxane (POSS) Copolymers. Macromolecules 40(3), 544 (2007).

[b18] Zheng L., Farris R. J. & Coughlin E. B. Novel Polyolefin Nanocomposites: Synthesis and Characterizations of Metallocene-Catalyzed Polyolefin Polyhedral Oligomeric Silsesquioxane Copolymers. Macromolecules 34, 8034 (2001).

[b19] Fang Y. F. & Chen S. Facile and Quick Synthesis of Poly(N-methylolacrylamide)/Polyhedral Oligomeric Silsesquioxane Graft Copolymer Hybrids via Frontal Polymerization. J. Polym. Sci., Part A: Polym. Chem. 47, 1136 (2009).

[b20] Lagaly G. Introduction: From Clay Mineral-Polymer Interactions to Clay Mineral-Polymer Nanocomposites. Appl. Clay Sci. 15(1–2), 1–9 (1999).

[b21] Levchik S. V. & Weil E. D. Overview of Recent Developments in the Flame Retardancy of Polycarbonates. Polym. 54, 981–998 (2005).

[b22] Sánchez-Soto M., Schiraldi D. A. & Illescas S. Study of the Morphology and Properties of Melt-mixed Polycarbonate–POSS Nanocomposites. Eur. Polym. J. 45, 341–352 (2009).

[b23] Zhang Y., Lee S., Yoonessi M., Liang K. & Pittman C. U. Phenolic Resin Trisilanolphenyl Polyhedral Oligomeric Silsesquioxane (POSS) Hybrid Nanocomposites: Structure and Properties. Polym. 47, 2984–96 (2006).

[b24] Zheng J., Kumar S., Iyer S., Schiraldi D. A. & Gonzalez R. I. Reinforcement of Polyethylene Terephthalate Fibers with Polyhedral Oligomeric Silsesquioxanes (POSS). High Performance. Polym. 17, 403–24 (2005).

[b25] Song L., He Q. L., Hu Y., Chen H. & Liu L. Study on Thermal Degradation and Combustion Behaviors of PC/POSS Hybrids. Polym. Deg. Stability 93(3), 627–639 (2008).

[b26] Prest W. M. & Luca D. J. The Origin of the Optical Anisotropy of Solvent Cast Polymeric Films. J. Appl. Phys. 50(10), 6067–6071 (1979).

[b27] Prest W. M. & Luca D. J. Compression of Two Polymercoated Surfaces in Poor Solvents. J. Chem. Phys. 105, 706 (1996).

[b28] Prest W. M. & Luca D. J. The Alignment of Polymers During the Solvent Coating Process. J. Appl. Phys. 51, 5170 (1980).

[b29] Wilson R. . Influence of Clay Content and Amount of Organic Modifiers on Morphology and Pervaporation Performance of EVA/Clay Nanocomposites. S. Ind. Eng. Chem. Res. 50(7), 3986–3993 (2011).

[b30] Siegmann A. & Geil P. H. Crystallization of Polycarbonate from the Glassy State. Part II. Thin Films Melted and Quenched. J. Macromole. Sci. Part B: Phys. 4, 273–291 (1970).

[b31] Egami T. & Billinge S. J. L. Underneath the Brag Peaks, Structure Analysis of Complex Material Ch. 2, 25–54 (Pergamon, Newyork, 2003).

[b32] Laulhé C., Hippert F., Bellissent R., Simon A. & Cuello G. Local Structure in BaTi_1−x_ZrxO_3_ Relaxors from Neutron Pair Distribution Function Analysis. J. Phys. Rev. B 79, 064104 (2009).

[b33] Bandyopadhyay J. & Ray S. The Quantitative Analysis of Nano-clay Dispersion in Polymer Nanocomposites by Small-angle X-ray Scattering. Polym. 51, 1437–1449 (2010).

[b34] Garbauskas M. F. Handbook of Polycarbonate Science and Technology Ch. 13, 293–302 (Marcel Dekker, 2000).

[b35] Pratt G. J. & Smith M. J. A. Dielectric Spectroscopy of Bisphenol-A Polycarbonate and Some of Its Blends. Chapter 10. ACS Symposium Series, American Chemical Society, Washington, DC, 144–157 (1999).

[b36] Landry C. J. T. & Henrichs P. M. The Influence of Blending on the Local Motions of Polymers: Studies Involving Polycarbonate, Poly (methy1 methacrylate), and a Polyester. Macromolecules 22, 2157–2166 (1989).

[b37] Porter R. S. & Johnson J. F. The Entangle Concept in Polymer System. Chem. Rev. 66, 1–27 (1966).

[b38] Stroble G. The Physics of Polymer: Concept for Understanding Their Structure and Behavior Vol. 3 (Springer-Verlag Berlia, Heidelberg 2007).

[b39] Men Y. . Structural Changes and Chain Radius of Gyration in Cold-Drawn Polyethylene after Annealing: Small and Wide-Angle X-ray Scattering and Small-Angle Neutron Scattering, J. Phys. Chem. B 109, 16650–16657 (2005). 16853118 10.1021/jp052723g

[b40] Zheng L. . Polymer Nanocomposites through Controlled Self-Assembly of Cubic Silsesquioxane Scaffolds. Macromolecules 37(23), 8606–8611 (2004).

[b41] Zheng L., Waddon A. J., Farris R. J. & Coughlin E. B. X-ray Characterizations of Polyethylene Polyhedral Oligomeric Silsesquioxane Copolymers. Macromolecules 35(6), 2375–2379 (2002).

[b42] Mehendru P. C., Jain K. & Agarwal J. P. High Temperature Relaxations of Polycarbonate Thin Films. J. Phys, D. Appl. Phys. 13, 1497–1501 (1980).

[b43] Utracki L. A. & Simha R. Statistical Thermodynamics Predictions of the Solubility Parameter. Polym. Int. 53(3), 279–286 (2004).

[b44] Utracki L. A. Statistical Thermodynamics Evaluation of Polymer–Polymer Miscibility J. Polym. Sci. Part B: Polym. Phys. 42, 2909–2915(2004).

[b45] Jang B. N., Wang D. & Wilkie C. A. Relationship between the Solubility Parameter of Polymers and the Clay Dispersion in Polymer/Clay Nanocomposites and the Role of the Surfactant. Macromolecules 38(15), 6533–6543 (2005).

[b46] Yildirim E. & Yurtsever M. A. Comparative Study on the Efficiencies of Polyethylene Compatibilizers by using Theoretical Methods. J. Polym. Res. 19, 9771–7 (2012).

[b47] Hao N., Böhning M. & Schönhals A. Dielectric Properties of Nanocomposites Based on Polystyrene and Polyhedral Oligomeric Phenethyl-Silsesquioxanes. Macromolecules 40(26), 9672–9 (2007).

[b48] Qian Y., Wei P., Jiang P., Zhao X. & Yu H. Synthesis of a Novel Hybrid Synergistic Flame Retardant and its Application in PP/IFR. Polym. Deg. Stability 96, 1134–1140 (2011).

[b49] Sahoo B. N. & Balasubramanian K. Photoluminescent Carbon Soot Particles Derived from Controlled Combustion of Camphor for Superhydrophobic Applications. RSC Adv. 4, 11331 (2014).

[b50] Katiyar N. & Balsubramanian K. Thermal modelling of hybrid composites of nano cenosphere and polycarbonate for a thermal protection system. RSC Adv. 4, 47529 (2014).

[b51] Gupta R. & Balasubramanian K. Hybrid caged nanostructure ablative composites of octaphenyl-POSS/RF as heat shields. RSC Adv. 5, 8757 (2014).

[b52] Sahoo B. N., Sabrish B. & Balasubramanian K. Controlled Fabrication of Non-fluoro polymer Composite Film with Hierarchically Nano Structured Fibers. Prog. Org. Coat. 77, 904–907 (2014).

[b53] Sahoo B. N., Balasubramanian K. & Sucheendran M. Thermally Triggered Transition of Superhydrophobic Characteristics of Micro- and Nanotextured Multiscale Rough Surfaces. J. Phys. Chem. C 119(25), 14201–14213 (2015).

[b54] Mishra R., Fu B. X. & Morgan S. E. J. Surface Energetics, Dispersion, and Nanotribomechanical Behavior of POSS/PP Hybrid Nanocomposites. Polym Sci. Part B: Polym Phys. 45, 2441–2455 (2007).

[b55] Cassie A. B. D. & Baxter S. Wettability of Porous Surfaces. Trans Faraday Soc. 40, 546–551 (1944).

[b56] Wenzel R. N. Resistance of Solid Surfaces to Wetting by Water. Ind. Eng. Chem. 28, 1988–1994 (1936).

[b57] Giacomello A., Meloni S., Chinappi M. & Casciola C. M. Cassie-Baxter and Wenzel States on a Nanostructured Surface: Phase Diagram, Metastabilities, and Transition Mechanism by Atomistic Free Energy Calculations. Langmuir 28(29), 10764−10772 (2012). 22708630 10.1021/la3018453

[b58] Gao L. & McCarthy T. J. Contact Angle Hysteresis Explained. Langmuir 22, 6234–6237 (2006). 16800680 10.1021/la060254j

[b59] Gao L. & McCarthy T. J. How Wenzel and Cassie Were Wrong. Langmuir 23(7), 3762–3765 (2007). 17315893 10.1021/la062634a

[b60] Gao L. & McCarthy T. J. Reply to “Comment on How Wenzel and Cassie Were Wrong by Gao and McCarthy’. Langmuir 23(26), 13243 (2007). 10.1021/la7022117 18001069

[b61] Gao L. & McCarthy T. J. Wetting 101°. Langmuir 25(24), 14105–14115 (2009). 19627073 10.1021/la902206c

